# Impact of the progressive uptake of pneumococcal conjugate vaccines on the epidemiology and antimicrobial resistance of invasive pneumococcal disease in Gipuzkoa, northern Spain, 1998–2022

**DOI:** 10.3389/fpubh.2023.1238502

**Published:** 2023-08-31

**Authors:** Ayla Manzanal, Diego Vicente, Marta Alonso, Nekane Azkue, Maria Ercibengoa, José María Marimón

**Affiliations:** ^1^Microbiology Department, Osakidetza Basque Health Service, Donostialdea Integrated Health Organization, San Sebastián, Spain; ^2^Department of Preventive Medicine, University of the Basque Country (UPV/EHU), San Sebastián, Spain; ^3^Infectious Diseases Area, Respiratory Infection and Antimicrobial Resistance Group, Biodonostia Health Research Institute, San Sebastián, Spain

**Keywords:** pneumococcal conjugate vaccines, invasive pneumococcal disease, non-vaccine serotypes, pneumococcal antibiotic resistance, SARS-CoV-2 preventive measures

## Abstract

**Objectives:**

To analyze the impact of pneumococcal conjugate vaccines (PCVs) on the incidence of invasive pneumococcal diseases (IPDs) and pneumococcal antibiotic resistance in Gipuzkoa, northern Spain for a 25 years period.

**Methods:**

All cases of IPD confirmed by culture between 1998 and 2022 in a population of around 427,416 people were included. Pneumococci were serotyped and antimicrobial susceptibility was assessed by the EUCAST guidelines.

**Results:**

Overall, 1,516 *S. pneumoniae* isolates were collected. Annual IPD incidence rates (per 100,000 people) declined from 19.9 in 1998–2001 to 11.5 in 2017–19 (42.2% reduction), especially in vaccinated children (from 46.7 to 24.9) and non-vaccinated older adult individuals (from 48.0 to 23.6). After PCV13 introduction, the decrease in the incidence of infections caused by PCV13 serotypes was balanced by the increase in the incidence of non-PCV13 serotypes. In the pandemic year of 2020, IPD incidence was the lowest: 2.81. The annual incidence rates of penicillin-resistant isolates also decreased, from 4.91 in 1998–2001 to 1.49 in 2017–19 and 0.70 in 2020. Since 2017, serotypes 14, 19A, and 11A have been the most common penicillin-resistant types. The incidence of erythromycin-resistant strains declined, from 3.65 to 1.73 and 0.70 in the same years.

**Conclusion:**

PCV use was associated with declines in the incidence of IPD and the spread of non-vaccine serotypes, that balanced the beneficial effect off PCV13, some of them showing high rates of antibiotic resistance.

## 1. Introduction

The introduction of the heptavalent pneumococcal conjugate vaccine (PCV7) at the beginning of the 21st century has changed the epidemiology of invasive pneumococcal disease (IPD). This vaccine demonstrated its efficacy in preventing IPD caused by vaccine serotypes but also showed protection against pneumococcal pneumonia and otitis in vaccinees (1). PCV7 also had other beneficial effects such as decreasing the rates of antibiotic-resistant IPD and creating herd protection against pneumococcal infections in non-vaccinees due to their deleterious effect on carriage and transmission among vaccine serotypes (2). Subsequently, a 13-valent PCV (PCV13) was introduced, showing protection against the original seven and the additional six serotypes included (3, 4).

The beneficial effects of PCVs have been, however, partially offset by the appearance and spread of non-vaccine serotypes, which have significantly increased the incidence of IPD as well as resistance, with the emergence of several antibiotic-resistant serotypes (5, 6). This has led to the recent introduction of PCV15 and PCV20, covering more serotypes and recommended for adult vaccination.

At the beginning of 2020, the SARS-CoV-2 pandemic occurred and many non-pharmaceutical interventions (NPI) as home lockdown, social distancing, the use of face masks and handwashing among others were adopted by governments to slow the spread of this coronavirus (7). In many countries, these containment measures have been associated with significant reductions in invasive diseases caused by other respiratory pathogens typically transmitted via respiratory droplets, such as *Streptococcus pneumoniae*, *Haemophilus influenzae*, and *Neisseria meningitidis*, likely as a consequence of a reduction in their transmission (8). One way to measure the efficacy of pneumococcal conjugate vaccines is to analyze their impact on IPD. In Spain, IPD is not a mandatory notifiable disease, and the number of cases reported in the ECDC annual surveillance reports cover 80% of the national population according to estimates by the National Center for Epidemiology (9, 10).

In Gipuzkoa, PCV7 was introduced in 2001 for vaccination in a private market (non-subsidized), with progressive children uptake up to 40–60% (11). In 2010, PCV7 was replaced by PCV13 which in 2016 was included in the children vaccination schedule of the public health service with coverages over 95%. In a previous study, we analyzed the effect of PCV7 in young children and the older adult in our region (12). In the present study, and before the introduction of PCV20, we have analyzed the effects of PCV13 on IPD and the antibiotic resistance of invasive isolates as well as the effect of the SARS-CoV-2 pandemic on IPD.

## 2. Materials and methods

The study was conducted between January 1998 and December 2022 at the Donostia University Hospital (DUH), which has around 1,000 beds and is located in Donostia-San Sebastian, capital city of the province of Gipuzkoa, Basque Country, northern Spain. The overall population of Gipuzkoa has increased over the 24 years study period from 673,563 inhabitants in 2001 to 720,458 in 2020. The population included in the study was the entire population served by DUH, representing approximately 59% of the total population of the province each year. All incidence data in this study are expressed as cases per 100,000 population/year.

### 2.1. Case definition

A case of IPD was defined as the isolation of an *S. pneumoniae* from a sterile site: blood, cerebrospinal fluid, pleural fluid, peritoneal liquid or mastoiditis. All cases of IPD diagnosed by microbiological culture were included in the study. Pneumococcal isolates were identified by optochin susceptibility and bile solubility tests. Serotyping was performed by the Quellung reaction between 1998 and 2009, by multiplex-PCR between 2010 and 2017 (13) and using the S. PneumoStrip test since 2017 (14). The serotype of all isolates typed by the two molecular techniques was confirmed by the Quellung reaction.

Serotypes were grouped into five categories:

1. all serotypes
2. PCV7 serotypes: serotypes 4, 6B, 9 V, 14, 18C, 19F, and 23F
3. PCV13 additional serotypes (PCV13-AS): serotypes 1, 3, 5, 6A, 7F, and 19A
4. PCV13 serotypes: serotypes 1, 3, 4, 5, 6A, 6B, 7F, 9 V, 14, 18C, 19A, 19F, and 23F
5. non-vaccine serotypes (NVTs) or non-PCV13 serotypes

Antimicrobial susceptibility testing was carried out prospectively during all the study for clinical purposes by the broth microdilution method. Due to historical variations in the criteria for interpreting antibiotic MICs (15), the most recent EUCAST clinical breakpoints were applied to isolates from all the periods of the study (16).

### 2.2. Statistical analysis

Age adjusted annual incidence trends were analyzed using the Poisson regression model using the SPSS v.8.0. For comparison of resistance rates, the Chi square test or the Fisher test when appropriated were used. A *p* value <0.05 was establish as statistically significant.

## 3. Results

### 3.1. Isolate collection

**Figure 1:**
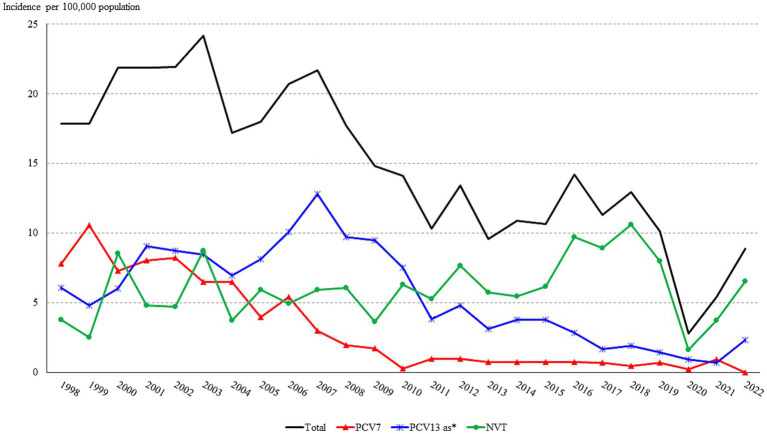
Trend in the annual incidence (cases per 100,000 population) of IPD in Gipuzkoa, northern Spain (1998–2020). *PCV13-AS: PCV13 additional serotypes (serotypes 1, 3, 5, 6A, 7F, and 19A).

Between 1998 and 2022, a total of 1,516 *S. pneumoniae* isolates causing invasive disease were collected: 155 (10.2%) in children younger than 5 years, 45 (3%) in children aged 5–14 years, 622 (41%) in adults aged 15–64 years and 694 (45.8%) in adults older than 64 years. The annual average incidence over the 25 years study was 14.7, decreasing from 17.9 cases in 1998 to 8.9 in 2022 (test for trend *p* < 0.001) (Figure 1). In March, April and May 2020, no cases of IPD were observed. Excluding 2020, 2021, and 2022, years of the SARS-CoV-2 pandemic, the year with the highest incidence was 2003 with 24.2 cases and the one with the lowest was 2013, with an incidence of 9.6.

Following the introduction of the PCV7 in 2001, the incidence of IPD decreased from more than 20 to around 10 in 2010. Subsequently, despite the introduction of the PCV13 in mid-2010, the overall incidence of IPD remained between 10 and 15 until 2020, the year in which we observed a large drop in the incidence. In 2021 and 2022 incidence progressively raised again, reaching 8.9 cases in 2022.

**Figure 2:**
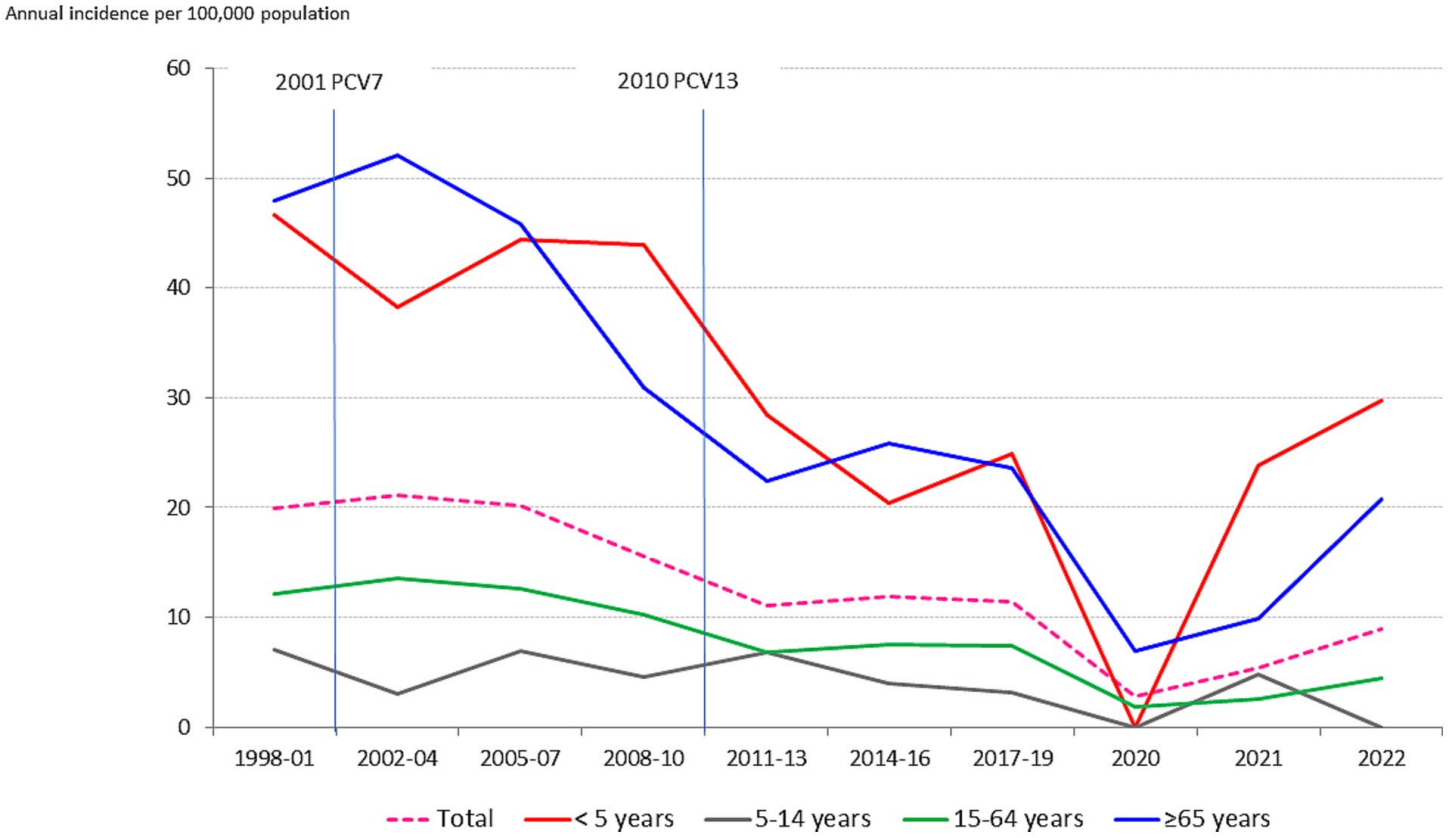
Annual incidence (cases per 100,000 population/year) of IPD by age group in Gipuzkoa, northern Spain (1998–2021).

These changes, however, differed by age group (Figure 2). The highest incidence rates of IPD were observed in children younger than 5 years, with an annual average of 34.15 (range 65.9 in 2008 to 0 in 2020), followed by older adults (average 31.9, range 60.3 in 2003 to 6.93 in 2020) (Table 1).

**Table 1:** Age group incidence (cases per 100,000 population/year) of IPD in Gipuzkoa, northern Spain (1998–2021) and specific 2020, 2021, and 2022 incidence data.

IPD	<5 years	5–14 years	15–64 years	≥65 years	Total
Average	34.15	4.60	9.3	31.9	14.69
Maximum (year)	65.93 (2008)	9.38 (2000)	16.05 (2003)	60.34 (2003)	24.15 (2003)
Minimum (year)*	11.13 (2015)	0 (2015 and 2019)	5.18 (2013)	16.55 (2014)	9.58 (2013)
2020	0	0	1.87	6.93	2.81
2021	23.81	4.82	2.61	9.89	5.38
2022	29.76	0.00	4.48	20.78	8.89

* Excluding 2020, 2021 and 2022.

From the pre-PCV period (1998–2001) to the last post-PCV13 period (2017–2019), the overall incidence decreased 43% (Table 2). This decrease was most pronounced in 5- to 14-year-olds, followed by those aged >65 years and < 5 years. Despite reductions in incidence in previous periods, in 2020 the decreases were nearly twice as large for all age groups.

**Table 2:** Average IPD incidence (cases per 100,000 population/year) and percent decrease in incidence by age group, Gipuzkoa, northern Spain (1998–2020).

Age groups	1998–2001 incidence	2017–2019 incidence	Decrease	2020 incidence	Decrease 2020 vs 2017–2019
<5 years	46.7	24.9	46.7%	0.0	100%
5–14 years	7.0	3.2	54.3%	0.0	100%
15–64 years	12.0	7.4	38.3%	1.9	74.3%
≥65 years	48.0	23.6	50.8%	6.9	70.8%
Total	19.8	11.3	42.9%	2.8	75.2%

### 3.2. Serotype distribution

**Figure 3:**
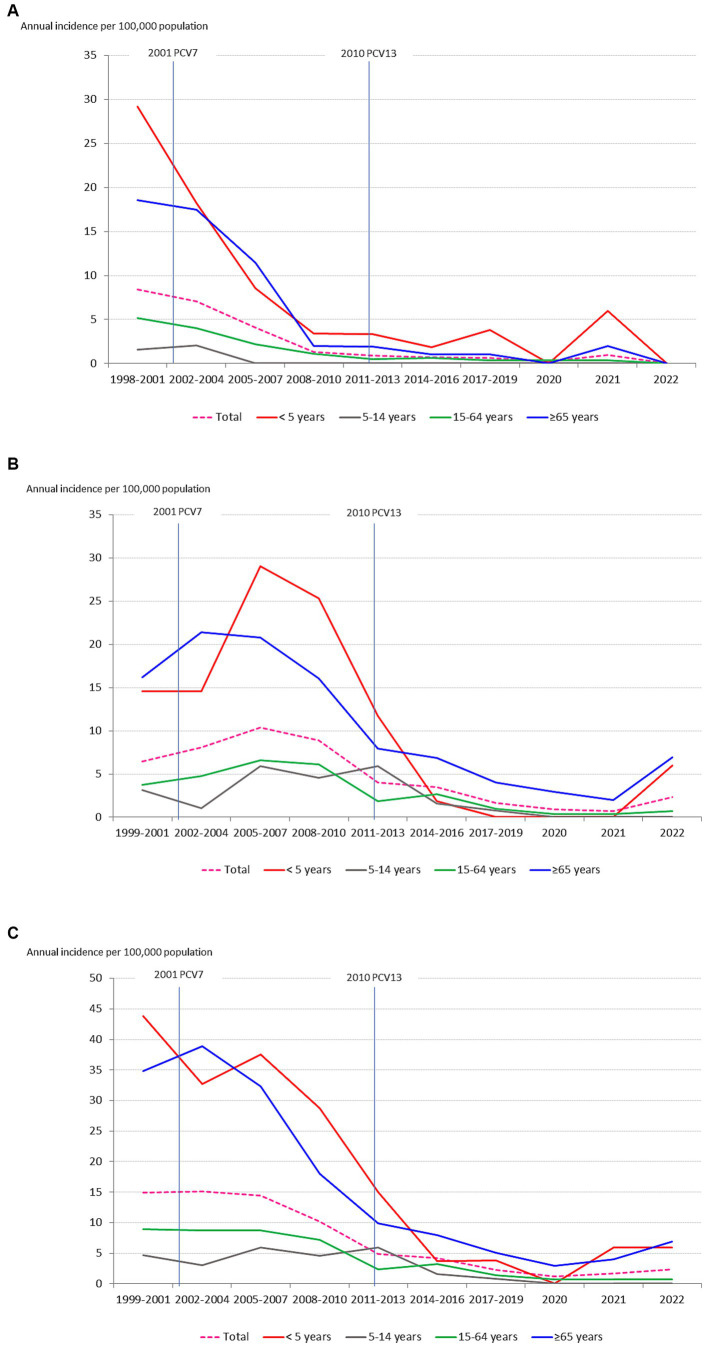

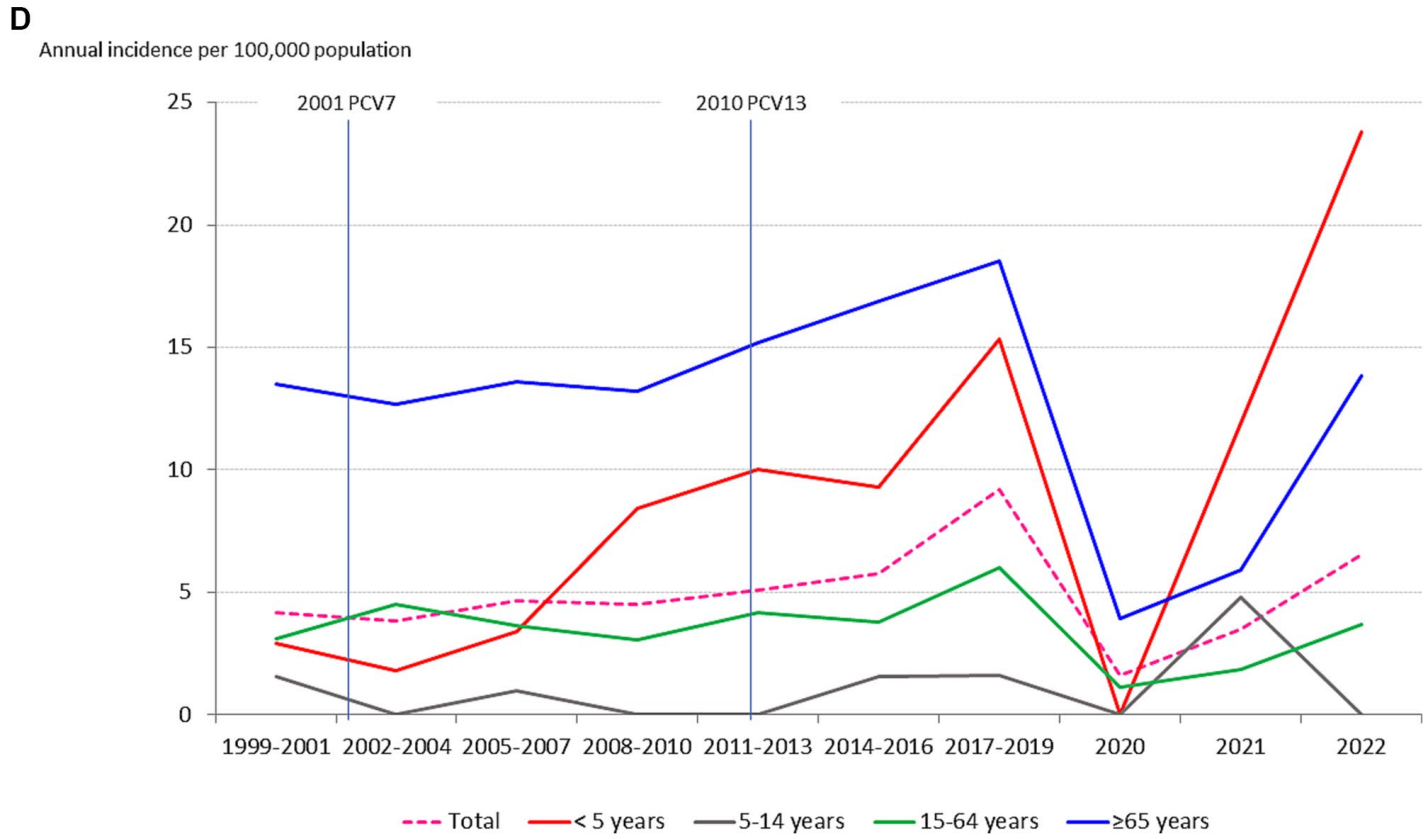
Changes in the incidence (cases per 100,000 population/year) of IPD serotypes over time by age group: **(A)** PCV7 serotypes; **(B)** PCV13 additional serotypes; **(C)** PCV13 serotypes; **(D)** NVTs (non-PCV13 serotypes).

The overall incidence of PCV7 serotypes decreased continuously since the introduction of PCV7 (*p* < 0.001) (Figure 3A). Contrarily, the incidence of PCV13-AS increased since 2001 mainly due to serotype 19A, which increased from 1.26 to 2.67 between 1998–2001 and 2008–2010, though it has decreased since then (Figure 3B) reaching an incidence of 0.70 in 2022. The incidence of PCV13 serotypes began to decrease from its maximum of 15.11 in 2002–04 due to a decrease in PCV7 serotypes and later from 2011–13 due to a decrease in PCV13-AS (Figure 3C). The incidence of serotype 3, which was analyzed individually, did not vary between 1999 and 2010, with incidence rates remaining above 2.0 (range 2.08–2.30), but then progressively decreased reaching the lowest incidence (1.02) in the period 2017–2019 to increase again to 1.64 in 2022.

The incidence of NVTs has steadily increased since the introduction of PCV7, but especially since 2014–2016 (Figure 3D), mainly due to increases in serotype 8 (2017–2019 incidence of 2.04), and to a lesser extent, in serotype 22F (2014–2016 incidence of 1.11), 12F (2017–2019 incidence of 0.94) and many other NVTs since 2017. In 2014–16, the incidence of NVTs was 5.76, this increasing to 9.18 in 2017–19, when the incidence of PCV13 serotypes was 2.28. The incidence of NVTs dramatically decreased during 2020 to 1.64 but began to increase again in 2021 reaching 6.55 in 2022.

For the last 5 years (2018–2022), the proportion of *S. pneumoniae* serotypes included in the new PCV20 vaccine (PCV13 serotypes plus serotypes 8, 10A, 11A, 12F, 15B, 22F, and 33F) was 64.9% (111/171 cases of IPD), with highest rates among adults 15–64 years old (76.9%) and adults ≥65 years old (62.2%). On the other hand, the overall proportion of IPD caused by PCV13 serotypes was 25.1% (43/171), 24.6% among adults 15–64 years old and 26.8% among adults ≥65 years old. In the same period 2018–22, the proportion of serotypes included in the pneumococcal polysaccharide vaccine 23-valent (PPV23) represented 71.3% of IPD infections, with a considerable increase in infections caused by serotype 12F (13 cases in 2018–2022 compared to 8 cases in 2013–2017 and 4 cases in 2008–2012).

Since 2011, the vaccination status of the 25 children <15 years old with IPD caused by PCV13 serotypes was checked. There were 11 with an infection caused by a PCV13-AS serotype that were too young at the moment of infection to have taken vaccination or were too old and had been vaccinated with PCV7. Of the remaining 14 patients, in 2 the vaccination status was unknown, 10 were not vaccinated and 2 were vaccinated with PCV13: a 2 years old boy great premature with a meningitis caused by a serotype 19F and a 2 years old girl with a mastoiditis also caused by a 19F serotype.”

### 3.3. Antibiotic resistance

**Figure 4:**
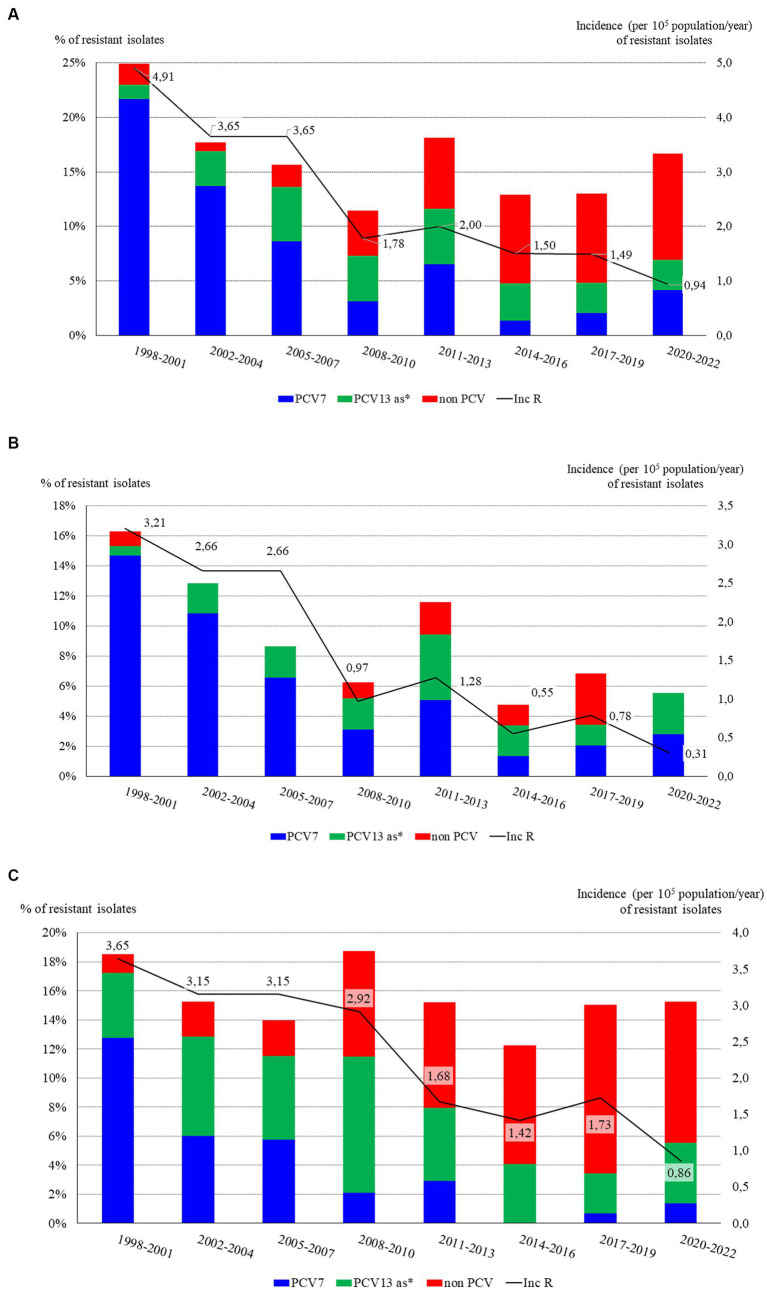

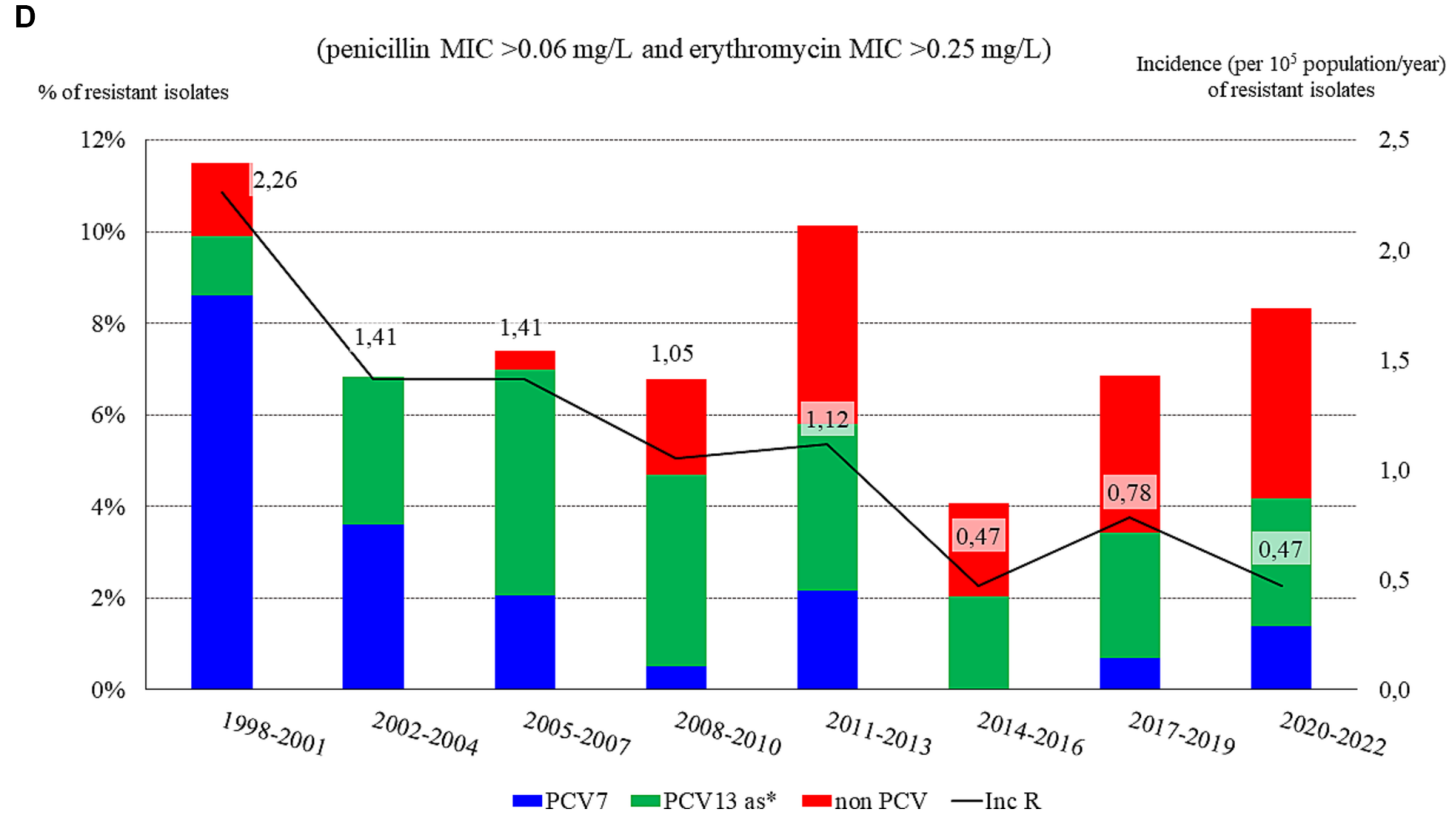
Time distribution of antibiotic resistance rates (bars) and incidence (line) of resistant isolates causing IPD in Gipuzkoa, northern Spain, 1998–2021: **(A)** Penicillin resistance; **(B)** Cefotaxime resistance; **(C)** Erythromycin resistance; **(D)** Multidrug resistance (MDR, i.e., resistance to both penicillin and erythromycin). *PCV13 as: PCV13 additional serotypes (serotypes 1, 3, 5, 6A, 7F, and 19A).

The overall incidence of penicillin resistant isolates progressively decreased from 4.91 cases in 1998–2001 to 0.94 in 2020–22 (chi square for trend 76.3, *p* < 0.001) due to a decrease in the incidence of IPD caused by PCV7 serotypes until 2010 and of serotype 19A since then (Figure 4A). Penicillin resistance (MIC >0.06 mg/L) rates decreased from 24.9% in the pre-PCV7 period (1998–2001) to 11.5% in 2008–2010 (*p* < 0.001). After 2010, penicillin resistance rates increased up to 16.7% in 2020–21 (*p* = 0.26), because of the increase in the proportion of serotype 19A and NVT-resistant isolates. Only 18 (1.2%) isolates were found to be highly resistant to penicillin (MIC >2 mg/L), of which 11 were serotype 14.

The incidence of IPD caused by amoxicillin-resistant isolates (MIC >1 mg/L) decreased over time, from 1.89 in 1988–2001 to 0.16 in 2020–21 (*p* < 0.001), mostly due to the decrease in the incidence of PCV7 isolates, which represented 78% (69 isolates) of the 88 amoxicillin-resistant isolates. Additional serotypes in PCV13 and NVTs accounted for 10.2% (9 isolates) and 11.4% (10 isolates) of amoxicillin-resistant isolates, respectively.

As happened with penicillin and amoxicillin, the incidence of cefotaxime resistant isolates (MIC >0.5 mg/L) gradually decreased from 3.21 to 0.31 (*p* < 0.001), due to the high decrease in the incidence of resistant PCV7serotypes (Figure 4B). In 2017–19 there were 10 cefotaxime-resistant isolates (rate 6.8%), of which 4 were NVTs, 3 being serotype 11A. Considering the cefotaxime resistance breakpoint for infections other than meningitis (MIC >2 mg/L), only 3 isolates were cefotaxime-resistant along the study: two serotype 14 and one serotype 19A.

Contrarily to beta-lactam antibiotics, the rates of erythromycin resistance did not clearly decrease over time, ranging between 12.2% (2014–16) and 18.5% (1998–2001) (chi square for trend 1.11 *p* = 0.29) (Figure 4C). PCV7 serotypes predominated in the pre-PCV7 period (69% of the 58 erythromycin-resistant isolates) and PCV13-AS between 2002 and 2010 (45.4% of the 117 erythromycin-resistant isolates, 43 serotype 19A). From 2011–13 to 2020–22, NVTs gained prominence representing 63.9% of the 72 erythromycin-resistant isolates. The incidence of IPD caused by erythromycin-resistant isolates continuously decreased over time, attributable to decreases in the incidence of PCV7 isolates from 2002 to 2004, and PCV13-AS from 2011 to 2013 (for incidence trend *p* < 0.001). Of the 238 erythromycin-resistant isolates, 189 (79.4%) were also clindamycin resistant (MLS_B_ phenotype). Of these, 127 were serotypes included in the PCV13. The three most frequent clindamycin-resistant serotypes were 19A, 6B and 14 with 55 (29.1%), 27 (14.3%) and 13 (6.9%) isolates, respectively.

As with other antibiotics, the incidence of trimethoprim-sulfamethoxazole (SXT) resistant isolates progressively decreased, from 3.57 to 1.30 between 2005–07 and 2008–10. In the following periods, the incidence of SXT-resistant isolates causing IPD remained at low levels, always under 1.4, with the lowest, 0.86 in the last period 2020–2022. Most (76.5%) SXT-resistant isolates obtained between 1998 and 2010 were PCV7 serotypes, serotypes 14 and 9 V standing out. From 2017 to 2022, the proportion of SXT-resistant NVTs increased when 17 of the 28 (60.7%) SXT-resistant isolates were NVTs.

We started routine levofloxacin resistance testing in 2001. Taking into account the current EUCAST breakpoint for levofloxacin resistance (MIC >2 mg/L), only 11/1369 (0.8%) levofloxacin-resistant pneumococcal isolates were identified as the cause of IPD, 8 of them also being moxifloxacin-resistant (MIC >0.5). No isolates were found to be resistant to vancomycin (MIC >2 mg/L) or rifampicin (MIC >0.5 mg/L). Chloramphenicol susceptibility was systematically tested until the end of 2016. This testing found 29/1366 (2%) isolates to be chloramphenicol resistant (MIC >8 mg/L), 21 of them serotypes included in the PCV7and 18 (62.1%) isolated in the pre-PCV7 period 1998–2001.

In this study, isolates resistant to penicillin (MIC >0.06 mg/L) and erythromycin (MIC >0.5 mg/L) were considered multidrug resistant (MDR). The incidence of invasive infections caused by MDR isolates has progressively decreased due to a drop in PCV7 serotypes since 2002–04 and a less pronounced decrease in PCV13-AS since 2014–16 (Figure 4D).

The highest rates and incidences of MDR isolates were observed in the pre-PCV7 period mainly due to serotypes 6B, 14, 23F, and 19F. In the following three periods (2002–04 to 2008–10), PCV13-AS dominated: 23 serotype 19A and 5 serotype 6A of the 48 MDR isolates. In the last 3 periods, NVTs became more prominent: 11 of the 22 MDR isolates were NVTs, of which 3 were serotype 6C and 3 serotype 24B. Of the PCV13 MDR isolates, 7/11 were serotype 19A.

## 4. Discussion

Many studies have demonstrated that the incidence of IPD varies over time in the same region and between regions in similar periods of time (10, 17, 18). For instance, the average annual incidence rates of IPD in a systematic review of studies performed between 1990 and 2019 were 15.08 in Spain versus 2.56 in Italy (17). In our study, an annual average of 14.96 per 100,000 was observed, very similar to the aforementioned systematic review (17) but higher than the range for Europe between 2013 and 2017, from 4.8 per 100,000 in 2014 to 6.2 in 2017 (19). Assuming that the methods used are appropriate and have been correctly implemented, local studies have the limitation of low representativeness due to the small geographical area studied, but the advantage of being able to document all the cases detected, which is why they generally give higher levels of incidence (20). Local, as well as larger national or international studies, are needed to provide a balanced solution in surveillance studies (21).

Due to the high efficacy of PCV7 and the collective protection it confers, a decrease in the overall incidence of IPD was observed in all age groups, especially the older adult. The decrease in the vaccine serotypes that caused IPD in Gipuzkoa could have been the consequence of the progressive increase in the number of children who received the vaccine together with a high vaccine efficacy (22). As has been described in larger studies (23, 24), IPD caused by PCV7 serotypes nearly disappeared in our region, but an increase in the incidence of non-PCV7 serotypes, especially serotype 19A was also observed. The effect of serotype replacement has been observed since the beginning of the use of PCVs (25, 26).

Despite PCV13 introduction in 2010, the overall incidence of IPD did not decline to 2019. A similar plateau in the decline in the incidence of IPD was observed in the same years in an adult population in a multicenter Spanish study (27). Interpreting this as a poor efficacy of PCV13 or that its introduction was not a cost–benefit measure should be done with caution, as the incidence of IPD caused by PCV13 serotypes did gradually decrease, especially during 2017–19. However, this effect was offset by the increased incidence of NVTs, which in 2017–19 quadrupled that of PCV13 serotypes, mainly due to the increased incidence of serotype 8. The highest serotype replacement event after PCV13 introduction in comparison with PCV7 could in part be explained by the fact that PCV13-AS are in general more frequently found in carriers than the PCV7 serotypes (28). Similarly, a single serotype stood up among the others after the introduction of each conjugate vaccine: serotype 19A after PCV7 and serotype 8 after PCV13. In other places where PCV13 was introduced, similar decreases in PCV13 serotypes and increases in NVTs have also been documented (24, 29–31). In most of these studies, serotypes 8, 15A, 22F, and 24F were the most frequent post-PCV13 serotypes causing IPD, while the importance of serotype 3 varied. The role of serotype 8 as a leading cause of IPD has also been revealed in many studies and 8 was the most frequent serotype causing IPD in Europe in 2018 (9). In Spain, a constant increase in serotype 8 infections was observed from 2015, it becoming the most prevalent serotype causing IPD in adults in 2019, and ranking second after serotype 24F in children <5 years (10). In our region, and to a lesser extent than serotype 8, the incidence rates of serotypes 12F and 22F have also increased since the introduction of PCV13. These emerging serotypes (8, 12F, and 22F) are included in the new PCV20 vaccine, already licensed by the FDA for the prevention of IPD in adults (32). The introduction of PCV20 should be accompanied, however, by a strict epidemiological surveillance of IPD to verify that the replacement with non-vaccine serotypes observed after PCV13 usage is not repeated.

The use of PCVs in children was associated with a reduction in IPD although the most marked decline in IPD was observed between 2019 and 2020, when SARS-CoV-2 NPI took place. Notably, 2020 was the year with the fewest cases of IPD (only 12), 5 of them before the beginning of the lockdown in Spain on 14th March and no cases then being observed until the end of June 2020, showing the efficacy of anti-COVID NPI in preventing other respiratory infection diseases. This additional effect of SARS-CoV-2 preventive measures has been already described, not only for *S. pneumoniae* but also for other bacterial pathogens transmitted via respiratory droplets (8). Such NPI are unlikely to be adopted again in the future but have given us the opportunity to observe their effectiveness in preventing many other infectious diseases.

One limitation of our study is that the incidence has been analyzed continuously, although there were two cohorts of vaccinated children, one with PCV7 and another with PCV13. This approach could have biased the real impact of vaccines on the incidence of IPD in the different groups of age as, for instance, some children <5 years of age vaccinated with PCV7 will have been included in the group of children 5–14 years old since 2010 without have not received PCV13, and some children of the 5–14 years old having received the PCV13 will have been included in the group of non-vaccinated young adults (15–64 years old) since 2019.

Trends in antimicrobial susceptibility data were studied adopting the latest EUCAST recommendations for the entire study, to avoid erroneous interpretation of the data due to changing criteria (15). Rates of penicillin resistance declined from 24.9% in 1998–2001 to 11.5% in 2008–10, remaining between 13 and 18% since then. These rates are in the range of the resistance rates observed in other European countries after PCV13 introduction (33–35). In Spain, recent studies have shown a penicillin-resistance rate of 21.2% in 2015–16 in adults associated with serotypes 11A, 14, and 19A (27), somewhat lower than the rate of 36.1% observed in children <18 years between 2012 and 2016 (36).

The rates of cefotaxime resistance were lowest in 2014–16 (4.8%), though slightly increased again in 2017–19 (6.8%) to reach 11.4% in 2020–22. Other studies have also placed cefotaxime among the most active anti-pneumococcal drugs, with even lower rates of resistance, ranging from 0.5 to 7.1% (27, 34, 35). What is of concern, however, is the growing number of cases of IPD caused by beta-lactam-resistant NVTs, mainly serotype 11A.

Erythromycin resistance rates ranged between 12.2 and 18.8%, without a decline in rates but with a notable decrease in the incidence of erythromycin-resistant isolates over time, especially after the introduction of PCV7 and PCV13. These rates were higher than the 8.3% reported in 2012–13 in adults in Germany (34), similar to the 16.3% reported in 2015–16 in Spain (27), and lower than the 21.7% reported for isolates causing non-invasive pneumococcal pneumonia in adults in 2012–15 in Portugal (31) and the 27% reported in 2011–12 in France (35). Nearly 80% of the erythromycin-resistant isolates from Gipuzkoa showed the MLS_B_ phenotype and from 2017 to 2022 they have mainly been NVTs, without any serotype dominating. In other studies, erythromycin resistance has been associated with certain serotypes, such as serotypes 19A and 24F in Spain (27) and serotypes 19F, 19A, 6C, and 15A in Portugal (33).

The disappearance of resistance to chloramphenicol over time is noteworthy, declining from 5.8% in the pre-PCV7 period (1998–2001) to no resistant isolates in 2008–10. This reversal of resistance can be explained by the non-use of chloramphenicol in therapy due to its side effects related to plastic anemia, as well as the decrease in the number of infections caused by PCV7 serotypes, which showed more resistance to chloramphenicol. In countries where chloramphenicol has been widely used, increasing pneumococcal resistance has been observed (37).

Resistance to other antibiotics such as fluoroquinolones was extremely low, or nil as in the case of vancomycin and rifampicin. In general, despite the wide use of fluoroquinolones in respiratory infections, fluoroquinolone resistance has remained at low levels in Spain (38) and in other countries with high levels of resistance to other antibiotics (39).

Although, in general, the rates of antibiotic resistance have stabilized since 2011–13 in Gipuzkoa, the incidence of IPD caused by resistant isolates has gradually decreased, because of the reduction in IPD caused by vaccine serotypes, which were the ones that showed the highest rates of antibiotic resistance. In the USA, from 1988 to 2018, a decrease in the incidence of non-susceptible IPD was also observed, both in children <5 years and adults ≥65 years old, while the incidence of non-susceptible NVT IPD has increased in recent years mainly due to serotypes 35B, 33F, 22F, and 15A (40). Most studies report data on antibiotic resistance in percentages (rates), although the incidence of resistant isolates is probably a more accurate figure as it takes into account the total number of resistant isolates. For example, in Gipuzkoa, the percentages of resistance to penicillin, cefotaxime or erythromycin have not decreased since 2008–10; nonetheless, the incidence of resistant isolates has steadily decreased up to 2020–22.

In conclusion, in Gipuzkoa, northern Spain, the introduction of PCV7 in 2001 and PCV13 in 2010 has been followed by a 2-fold reduction in IPD in all ages. The herd protection effect observed in the non-vaccinated older adult, however, seems to have reached its limit in part due to the emergence of NVTs such as serotype 8. The adopted SARS-CoV-2 NPI coincided with the lowest incidences of IPD, suggesting that they are also excellent measures to prevent the spread of respiratory bacterial pathogens, as in this case, pneumococcal transmission.

The use of vaccines has also been accompanied by decreases in the incidence of IPD caused by antibiotic-resistant serotypes. Nonetheless, the emergence of NVTs with antibiotic resistance makes it necessary to update vaccines to cover a wider spectrum of serotypes as well as continue surveillance of virulent serotypes.

## Data availability statement

The raw data supporting the conclusions of this article will be made available by the authors, without undue reservation.

## Ethics statement

Ethical approval was not required for the studies involving humans because the study was retrospective and no relevant patient clinical information was included. Patient epidemilogical data were handled in accordance with Spanish data protection laws and regulations in force (Organic Law 3/2018, of December 5, on the Protection of Personal Data and guarantee of digital rights). The studies were conducted in accordance with the local legislation and institutional requirements. Written informed consent for participation was not required from the participants or the participants’ legal guardians/next of kin in accordance with the national legislation and institutional requirements because this retrospective study was performed on bacterial isolates.

## Author contributions

AM and JM participated in the design of the study and writing the draft paper. DV, MA, and NA participated in performing pneumococcal isolates in blood cultures and pleural fluids. JM, ME, and AM participated in pneumococcal serotyping and in collecting patient’s epidemiological and microbiological data. AM, DV, MA, NA, ME, and JM contributed in the analysis of results and to the final writing, and review of the article. All authors contributed to the article and approved the submitted version.

## Conflict of interest

The authors declare that the research was conducted in the absence of any commercial or financial relationships that could be construed as a potential conflict of interest.

## Publisher’s note

All claims expressed in this article are solely those of the authors and do not necessarily represent those of their affiliated organizations, or those of the publisher, the editors and the reviewers. Any product that may be evaluated in this article, or claim that may be made by its manufacturer, is not guaranteed or endorsed by the publisher.
